# Enzymatic reactions towards aldehydes: An overview

**DOI:** 10.1002/ffj.3739

**Published:** 2023-04-10

**Authors:** Lukas Schober, Hana Dobiašová, Valentina Jurkaš, Fabio Parmeggiani, Florian Rudroff, Margit Winkler

**Affiliations:** ^1^ Institute of Molecular Biotechnology Graz University of Technology Graz Austria; ^2^ Institute of Chemical and Environmental Engineering Slovak University of Technology Bratislava Slovakia; ^3^ Dipartimento di Chimica, Materiali ed Ingegneria Chimica “Giulio Natta” Politecnico di Milano Milan Italy; ^4^ Institute of Applied Synthetic Chemistry TU Wien Vienna Austria; ^5^ Area Biotransformations Austrian Center of Industrial Biotechnology Graz Austria

**Keywords:** aldehyde, aliphatic aldehydes, aromatic aldehydes, aryl‐aliphatic aldehydes, biocatalysis, enzymes, green leaf volatiles, vanillin

## Abstract

Many aldehydes are volatile compounds with distinct and characteristic olfactory properties. The aldehydic functional group is reactive and, as such, an invaluable chemical multi‐tool to make all sorts of products. Owing to the reactivity, the selective synthesis of aldehydic is a challenging task. Nature has evolved a number of enzymatic reactions to produce aldehydes, and this review provides an overview of aldehyde‐forming reactions in biological systems and beyond. Whereas some of these biotransformations are still in their infancy in terms of synthetic applicability, others are developed to an extent that allows their implementation as industrial biocatalysts.

## Graphical abstract

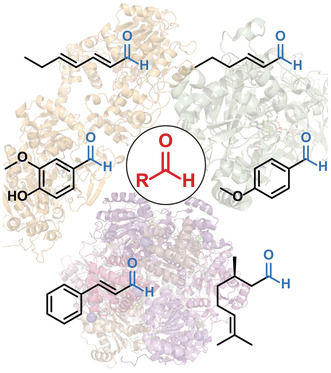

## 1 INTRODUCTION

Aldehydes are reactive compounds and can undergo chemical transformations to numerous other functional groups.^1^ The aldehyde is therefore an invaluable chemical multi‐tool to make all sorts of products.^2^ As final products, aldehydes find application in the flavour and fragrance sector, because they are often volatile with characteristic olfactory properties. The most abundant biomass‐derived aldehydes are furfural from cellulose, 5‐hydroxymethylfurfural (HMF, 5‐(hydroxymethyl)‐2‐furaldehyde) from hemicellulose, aromatic aldehydes like vanillin and syringaldehyde from lignin^3^ as well as short‐, medium‐ and long‐chain aldehydes from oil and fat‐derived (polyunsaturated) fatty acids. Lignin, for example, is a largely untapped resource, since nowadays it is primarily burnt to generate heat.^4^

The flavour and fragrance industry is interested in these kinds of molecules, and enzymatic and biotechnological processes towards ‘bio‐aldehydes’ are gaining relevance.^2^ Valuable compounds can be produced from renewable resources based, for example, on the degradation of lignin, that produces monolignols. Biotechnological production decreases the dependence on plant material, whose availability greatly varies with season, weather, political developments, need as food or feed and many factors more. While in the consumer's eye, natural ingredients are clearly favoured over chemically synthesised ingredients, biocatalysis,^5^ biotechnology and metabolic engineering may close gaps, especially for desirable compounds from rare and endangered species. The ‘naturality’ of ingredients is a richly facetted chapter.^6^ Whether an ingredient can be labelled ‘natural’ or not is a matter of definitions and regulations. Naturality is not easily measurable. It is clear that an apple is natural, but when this apple is processed to generate a flavour ingredient, it depends on each processing step and its associated auxiliaries whether the final ingredient can still be considered natural. Phrasing it in a very simplistic way, the ‘greenness’ of each processing step helps to preserve the naturality status of an ingredient. Mild and biobased methodologies play a key role in this context.

**SCHEME 1:**
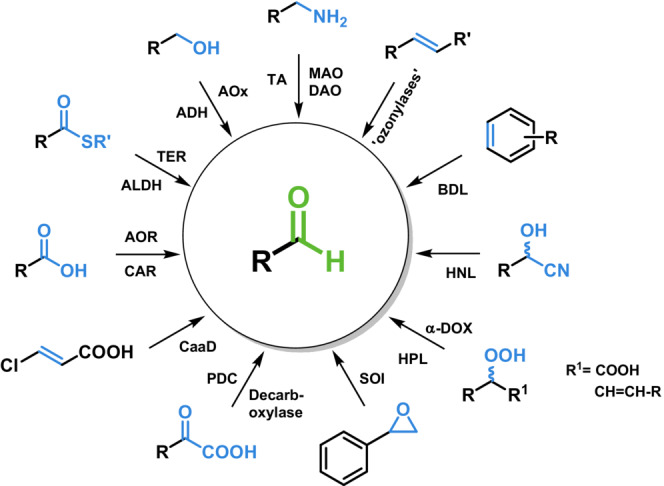
Enzymatic reactions to aldehydes. Aldehyde‐forming aldolases were omitted due to their need for aldehydes as substrates.

Nature is equipped with a portfolio of proteins with enzymatic activity to transform various precursor molecules to the respective aldehydes, and these are the focus of this review (Scheme 1).

## 2 ENZYME CATALYSED ALDEHYDE FORMATION

### 2.1 From carboxylic acids

Carboxylic acids are stable and abundant in biomass and they can be produced in good amounts by acidogenic fermentation.^7^ Volatile organic acids are, for example, accessible by anaerobic fermentation from organic wastes. A broad spectrum of fatty acids is available from fats and oils, including also waste streams from edible oil production. Such carboxylic acids are a stable and renewable pool of aldehyde precursors.

Two distinct enzyme families are known to reduce acids to aldehydes: carboxylic acid reductases (CARs) and aldehyde oxidoreductases (AORs).

#### 2.1.1 Aldehyde oxidoreductase

**SCHEME 2:**
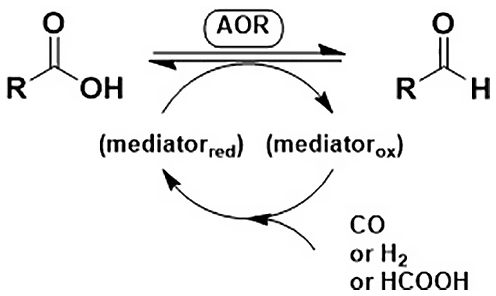
Aldehyde oxidoreductases AORs (EC 1.2.99.6) reduce short‐chain carboxylic acids.

Aldehyde oxidoreductases (EC 1.2.99.6) catalyse the reduction of a carboxylate to an aldehyde and the reverse reaction.^8^ AORs exhibit a relatively broad substrate spectrum, using short‐chain aliphatic aldehydes, branched‐chain aliphatic aldehydes and aromatic aldehydes and their corresponding acids (Scheme 2). AORs are oxygen sensitive, tungsten containing enzymes from bacterial and archaeal origin. They harbour Fe–S clusters and operate under strictly anaerobic conditions in the presence of redox mediators.^9^, ^10^ Due to this sensitivity, AORs have mainly been studied in the form of whole‐cell biocatalysts in their natural hosts (e.g. *Pyrococcus furiosus* DSM 3638) or as purified enzymes from their native organisms. In the strictly anaerobic natural hosts, further reduction of the emerging aldehyde to alcohols was predominant. Aldehyde:ferredoxin oxidoreductase, for example, reduces acetate to acetaldehyde via reduced ferredoxin as a redox carrier.^11^ In *P. furiosus*, hydrogen (H_2_) is oxidised by a hydrogenase enzyme and serves as a reducing equivalent.^12^ A major scientific challenge that—to the best of our knowledge—has not been solved so far is to functionally express AORs in a heterologous host such as *Escherichia coli* or yeast.

#### 2.1.2 Carboxylic acid reductase

**SCHEME 3:**
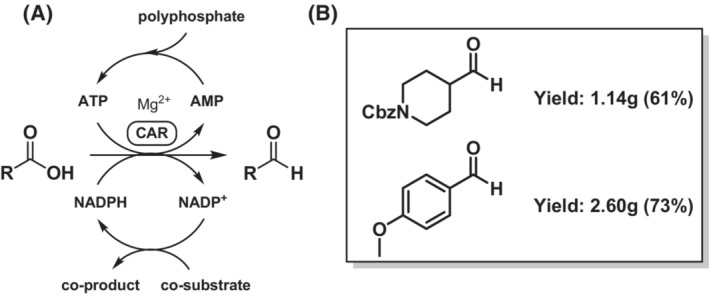
(A) Carboxylic acid reductase (CAR EC 1.2.1.30) mediated enzymatic aldehyde synthesis; for application in cell‐free systems, ATP and NADPH are recycled. (B) Isolated products form cell‐free aldehyde synthesis.

Carboxylic acid reductases (EC 1.2.1.30) also catalyse the single‐step reduction of a carboxylate to an aldehyde (Scheme 3). The reverse reaction has not been reported. CARs are relaxed regarding structural features of the acids they reduce and can cope with aliphatic, aromatic, heteroaromatic and aryl‐aliphatic mono‐ and di‐carboxylates. Halogen, hydroxy, methyl, methoxy and amino substitutions are tolerated well, in case they are two carbon atoms or more distant to the carboxylic acid moiety.^13^ Due to their broad product scope, CARs hold great promise as a tool for the synthesis of various aldehydes.

Acid activation is accomplished directly in the CAR enzyme by adenosine triphosphate (ATP), and the nicotinamide cofactor NADPH serves as the reductant (Scheme 3). These cofactors are required in stoichiometric amounts. When CARs are utilised as isolated enzymes, cofactor supply can be accomplished by catalytic amounts of ATP and NADPH and their recycling in vitro (Scheme 3). Well‐established oxidoreductase recycling systems are available for NADPH (e.g. glucose dehydrogenase/glucose). ATP recycling is typically accomplished with kinase enzymes at the expense of polyphosphate. The reader is referred to a recent review by Tavanti et al.^14^ for detailed information. New kinases are currently becoming available, as ATP recycling is in the research focus of both academia^15^, ^16^ and industry.^17^ For aldehyde synthesis, the in vitro strategy has been shown successful, for example, for the preparation of 4‐methoxy‐benzaldehyde (anisaldehyde)^17^ and *N*‐carbobenzoxylated 4‐formylpiperidine^18^ on a gram scale (Scheme 3). Vanillin can be produced in 2.86 g L^−1^ concentration using an *E. coli*‐based whole‐cell biocatalyst equipped with *Mycobacterium abscessus* CAR *Ma*CAR2,^19^ or as vanillyl‐glycoside on industrial scale in yeast.^20^

### 2.2 From thioesters

Thioesters are carboxylic acid derivatives which are formed in living systems in the course of the biosynthesis of fatty acids, polyketides and non‐ribosomal peptides, and other related metabolic processes. The thio‐donor in living systems is Coenzyme A (CoA), or the phosphopantetheine unit of CoA that is attached to protein. Also, proteinogenic cysteines may function as thio‐donors.

#### 2.2.1 Thioester reductase and thioester reductase domains in larger proteins

Thioester reductases (TERs) catalyse the NAD(P)H‐dependent reduction of thioesters to aldehydes. Acyl protein thioester reductases (EC 1.2.1.50, EC 1.2.1.80) produce long‐chain aldehydes from protein‐bound fatty acids. Fatty aldehydes are furthermore accessible through CoA‐bound fatty acids, which are reduced by fatty acyl‐CoA reductases (FARs, EC 1.2.1.42 and EC 1.2.1.B25). Short‐chain aldehydes as primary metabolites are the products of several enzyme classes which are typically named according to their natural product: acetaldehyde dehydrogenase (EC 1.2.1.10) produces acetaldehyde from acetyl‐CoA. Others act on CoA‐bound malonates (EC 1.2.1.18, EC 1.2.1.27 and EC 1.2.1.75), to deliver malonate‐semialdehyde or CoA‐bound succinate (EC 1.2.1.76), or glyoxylates (EC 1.2.1.17, EC 1.2.1.58), respectively.

Thioester reductases also occur as domains in multi‐domain proteins. One example thereof was outlined for CARs (chapter 2.1.2), in which the R‐domain is in fact catalysing thioester reduction. Similarly, NRPS and PKS with thioester reductase domains were reported to produce complex aldehydes^21^, ^22^ and amino aldehydes which eventually tend to condense to give pyrazines.^23^, ^24^ Thioester reductase domains may be promiscuous in the sense that not only the thioester delivered by its associated transthiolation domain is reduced, but also unbound surrogates, albeit with less efficiency.^25^

Notably, 3‐ketoacyl‐thioester reductase (EC 1.1.1.100) and 2‐enoyl (thioester) reductases (EC 1.3.1.10 and EC 1.3.1.38) do not produce aldehydes but act on other reducible groups in protein‐bound thioesters.

**SCHEME 4:**
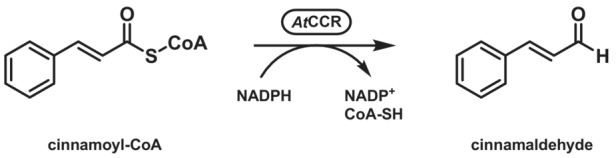
Last step of the biosynthetic pathway to cinnamaldehyde catalysed by cinnamoyl‐CoA reductase (CCR).

Due to the need for coenzyme A or a particular protein for thioester formation, thioester reductases are mostly targeted in metabolic engineering campaigns. Aldehydes themselves are rarely the desired product but appear as transient key components, for example, for the production of the corresponding alcohols.^26^ An exception is the synthesis of cinnamaldehyde, which is generated, for example, by a cinnamoyl‐CoA reductase from *Arabidopsis thaliana* (Scheme 4) as the final product.^27^

#### 2.2.2 Aldehyde dehydrogenase and aldehyde reductase

The thioester may also be formed by a proteinogenic cysteine. A recent publication showed that the adenosine monophosphate (AMP)‐anhydride of hydroxylated 2,2′‐bipyridine‐1‐carboxylate reacted with a C‐terminal cysteine of CaeB2, a protein with high similarity to NADPH‐dependent aldehyde dehydrogenases (ALDHs). CaeB2 hence catalysed the transthiolation of an AMP‐anhydride followed by NADPH‐dependent thioester reduction to 2,2′‐bipyridine‐1‐carbaldehyde.^28^

### 2.3 From primary alcohols

Alcohols are an abundant source of compounds from renewable sources, already widely used in many industries. Higher molecular weight alcohols (e.g. geraniol, cuminol, 2‐phenylethanol) are often isolated from plant material by steam distillation. Fatty alcohols are usually obtained from fats and waxes by base hydrolysis followed by reduction, but can also be produced by microbial fermentation.^29^

The most commonly used enzymes for the oxidation of alcohols are alcohol dehydrogenases (ADHs) and alcohol oxidases (AOxs). Peroxidases and oxygenases can also be used but have received little attention because of narrow substrate scope or low selectivity.^30^, ^31^

#### 2.3.1 Alcohol oxidase

**SCHEME 5:**
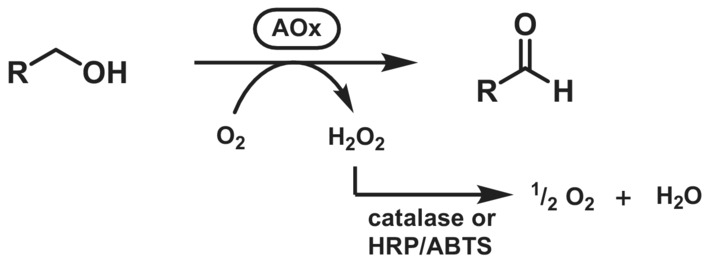
Oxidation of primary alcohols catalysed by alcohol oxidase (AOx).

Alcohol oxidases (EC 1.1.3.x) oxidise alcohols to aldehydes or ketones using O_2_ as a terminal electron acceptor. The reaction produces H_2_O_2_ as a side product. To avoid enzyme deactivation, H_2_O_2_ is usually removed in situ by an accessory enzyme: a catalase or a peroxidase. Thus, the net reaction becomes irreversible (Scheme 5). In addition, horseradish peroxidase (HRP) can be conveniently coupled with 2,2′‐azino‐bis(3‐ethylbenzothiazoline‐6‐sulfonic acid) (ABTS) as an electron acceptor in a spectrophotometric assay for screening purposes.^32^ Except for some cofactor‐lacking oxidases, electron transfer is mediated by a tightly bound redox‐active prosthetic group, usually a copper or FAD.^32^, ^33^ A major advantage of the AOx over ADH enzymes is the lack of dependence on the costly NAD(P)^+^ cofactor. The major drawbacks are their comparably lower abundance in nature and often they are of eukaryotic origin, making recombinant expression more challenging.

##### Copper radical alcohol oxidase

Copper radical alcohol oxidases (CRO‐AOxs) contain a single copper(I) ion bound in a shallow active site. Substrate oxidation involves the formation of a tyrosine radical that is stabilised by a cross‐linked tyrosine–cysteine residue and ultimately forms a metalloradical complex.^32^, ^34^

**SCHEME 6:**
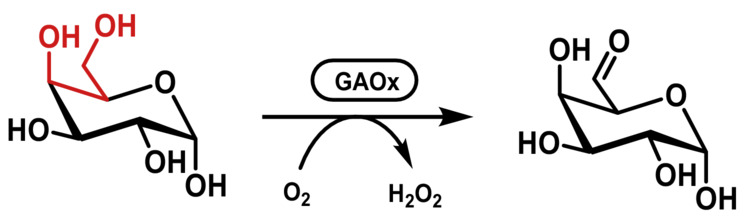
Oxidation of the primary alcohol of galactose by the galactose oxidase. The substrate recognition motif is highlighted in red.

The archetypal CRO‐AOx is galactose oxidase (GAOx, EC 1.1.3.9), whose natural activity is oxidation of the C‐6 OH group of d‐galactose to give the aldehyde d‐galacto‐hexodialdose (Scheme 6).^35^ The enzyme is highly regioselective and next to galactose itself, is able to oxidise galactose‐containing saccharides such as lactose. The recognition motif of the enzyme seems to be the C‐4 to C‐6 diol, as certain primary alcohols like, for example, the *N*‐acetylalcohol of *N*‐glycolylneuraminic acid (Neu5Gc) are also oxidised.

Galactose oxidase enzymes are applied for the functionalization of saccharides, as biosensors and as key enzymes in various enzymatic cascades designed for the transformation of HMF into furandicarboxylic acid (FDCA).^36^, ^37^, ^38^ In this context, (di)aldehydes are transitional intermediates. Overoxidation of aldehydes to carboxylates was observed as a side reaction of GAOx, and was also purposefully engineered into GAOx enzymes.^39^

Efforts to expand the substrate scope and increase the catalytic efficiency of GAOx have predominantly been carried out on the GAOx from the phytopathogenic fungus *Fusarium graminearum*, which was the first described and remains the most prominent member of the CRO‐AOx family. The substrate scope was expanded not only to other carbohydrates such as glucose,^40^ fructose,^41^ mannose and *N*‐acetylglucosamine,^42^ but also secondary alcohols,^43^ amino alcohols^44^ and benzyl alcohols.^39^ Notably, a highly evolved variant was developed for industrial biocatalytic cascade synthesis of the drug islatravir.^45^

In the last decade, new types of CRO‐AOx enzymes were found based on the structural similarity to the GOx, but with surprisingly low activity towards carbohydrates. Most of these enzymes were identified in the phytopathogenic genera *Colletotrichum* and *Fusarium*, and catalyse the oxidation of diverse activated and non‐activated aliphatic and aromatic alcohols—activity that classifies them either as specific alcohol oxidases (EC 1.1.3.13) or aryl alcohol oxidases (AAOx, EC 1.1.3.7).^46^, ^47^, ^48^, ^49^, ^50^, ^51^

**SCHEME 7:**
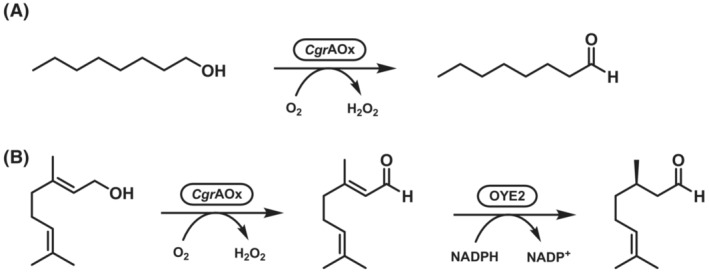
Primary alcohol oxidations catalysed by the CRO‐AOx from *Colletotrichum graminicola* (*Cgr*AOx). (A) Oxidation of octan‐1‐ol to octanal. (B) Oxidation of geraniol to geranial by *Cgr*AOx followed by C=C reduction by enoate‐reductase OYE2 to produce (*R*)‐citronellal.

Alcohol oxidases from *Colletotrichum graminicola* (*Cgr*AOx) and *Colletotrichum destructivum* (*Cde*AOx) were used for oxidation of C6 and C8 primary aliphatic alcohols into the corresponding fragrance aldehydes. Upon longer reaction times, overoxidation of >C6 substrates to corresponding carboxylic acids was observed and associated to the propensity of the aldehyde to undergo hydration and form a geminal diol. The presence of an electron‐withdrawing group increased aldehyde overoxidation (24% aldehyde hydrate formation in aqueous solution), while an electron‐donating group produced only aldehyde (0% hydrate formation). Enzyme inhibition by the long‐chain fatty aldehyde hydrates was also described.^50^, ^51^ A gram‐scale reaction using a crude preparation of *Cgr*AOx yielded 0.72 g of octanal (Scheme 7A).^51^ *Cgr*AOx was also applied in a sequential one‐pot cascade for oxidizing geraniol to geranial, which was subsequently reduced by an ene‐reductase to (*R*)‐ or (*S*)‐citronellal. A quantity of 62 mg of geraniol was converted to (*R*)‐citronellal with 98% conversion and 72% isolated yield (Scheme 7B).^52^

**SCHEME 8:**
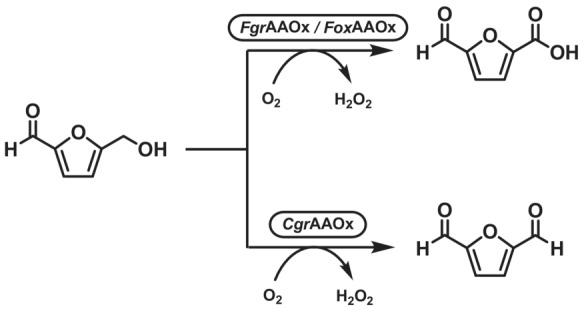
Oxidations of HMF catalysed by CRO‐AOx aryl alcohol oxidases (AAOx). The AAOx from *Fusarium* species (*Fgr*AAOx and *Fox*AAOx) overoxidize HMF to 5‐formyl‐2‐furoic acid (FFCA). The AAOx from *Colletotrichum graminicola* (*Cgr*AAOx) produces the di‐aldehyde 2,5‐diformylfuran (DFF) without overoxidation.

The overoxidation phenomenon does not only depend on the propensity of the aldehyde to form the aldehyde hydrate, but also on the ability of the specific enzyme to accommodate the geminal diol in the active site. For example, while the AAOx from *Fusarium* species overoxidizes HMF to 5‐formyl‐2‐furoic acid (FFCA), respectively,^48^ the AAOx from *C. graminicola* can chemoselectively oxidise HMF to 2,5‐diformylfuran (Scheme 8).^47^

##### Flavin‐dependent alcohol oxidase

Flavin‐dependent alcohol oxidases (FAD‐AOx; EC 1.1.3.13) contain a tightly, sometimes even covalently, bound flavin cofactor (generally FAD) that serves as the primary hydride acceptor. Substrate oxidation generates a reduced flavin which is re‐oxidised using molecular oxygen via a hydroxyperoxyflavin intermediate, generating H_2_O_2_. Often, the oxidation of primary alcohols does not stop at the aldehyde stage, but proceeds to the corresponding carboxylic acid.^32^

Based on the structural fold, these enzymes can be classified into two main families: the glucose‐methanol‐choline oxidase family (known as the GMC family) and the vanillyl‐alcohol oxidase family (known as the VAO family) which can further be divided based on the presence and number of covalent bonds present between the FAD cofactor and the protein part of the specific oxidase.^53^

Members of the GMC family include short‐chain alcohol oxidases (SCAOx), long‐chain alcohol oxidases (LCAOx) and aryl alcohol oxidases (AAOx). SCAOxs are key enzymes of methanol metabolism of methylotrophic yeasts such as *Komagataella phaffii* (*Pichia pastoris*). The AOx from *K. phaffii* has high activity towards short‐chain aliphatic alcohols (methanol, ethanol), but activity decreases with increasing chain length. However, the oxidation of longer chains (C6–C11) and aromatic alcohols is possible in biphasic systems.^54^, ^55^

An interesting enzyme belonging to the GMC family is the 5‐hydroxymethylfurfural oxidase from *Methylovorus* sp. strain MP688 (EC 1.1.3.47). The enzyme is highly selective in accepting only hydrated aldehydes, primary alcohols and thiols as substrates and at the same time strikingly promiscuous concerning the side chain. Except for HMF, it accepts various furanic, benzylic and cinnamic alcohols, with ring substituents of different sizes, polarities and charges, oxidizing them to carboxylic acids.^56^, ^57^

**SCHEME 9:**
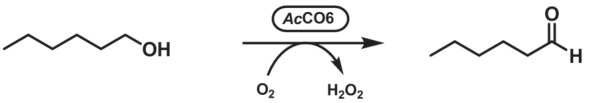
Oxidation of hexan‐1‐ol to hexanal catalysed by the sixfold variant of choline oxidase from *Arthrobacter chlorophenolicus* (*Ac*CO6).

In terms of aldehyde synthesis, overoxidation is undesirable, but it may be eliminated through enzyme engineering, as shown for the choline oxidase (CO, EC 1.1.3.17) from *Arthrobacter chlorophenolicus* which catalyses the two‐step oxidation of choline into trimethylglycine. Structure‐guided directed evolution of the *Ac*CO produced a six amino acid variant (*Ac*CO6) with much broader substrate specificity towards a range of primary alcohols and increased thermostability and solvent tolerance. Saturated alcohols with C6–C10 chain lengths and unsaturated alcohols were especially good substrates. Hexan‐1‐ol (100 mg) was completely converted to hexanal in 72% isolated yield (Scheme 9). Overoxidation was still detected with other substrates (e.g. cinnamyl alcohol) but could be minimized by applying a biphasic system.^58^ In a follow‐up study, oxidation of hexan‐1‐ol by *Ac*CO6 in continuous flow did lead to the overoxidation to the carboxylic acid when performed in buffer, but could be completely avoided by using neat solvent as the reaction medium.^59^

**SCHEME 10:**
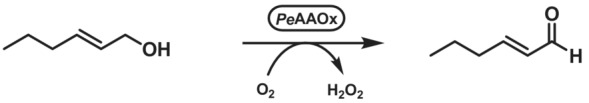
Oxidation of *trans*‐hex‐2‐en‐1‐ol to *trans*‐hex‐2‐enal catalysed by the aryl alcohol oxidase from *Pleurotus eryngii* (*Pe*AAOx).

Aryl alcohol oxidase from the fungus *Pleurotus eryngii* (*Pe*AAOx) was used for biocatalytic preparation of the green note aroma compounds *trans*‐hex‐2‐enal from *trans*‐hex‐2‐en‐1‐ol (Scheme 10). Initially, biocatalytic synthesis of 200 mg of *trans*‐hex‐2‐enal in a continuous flow reactor was reported, with turnover frequencies up to 38 s^−1^ and turnover numbers >300,000.^60^ A follow‐up study described a two‐liquid phase system approach using the substrate itself as the organic phase. *trans*‐Hex‐2‐enal accumulated over 14 days with multiple additions of *Pe*AAOx and catalase to 255 g L^−1^. Although the conversion was only 31%, the total turnover number of *Pe*AAOx reached >2.2 × 10^6^.^61^

**FIGURE 1:**
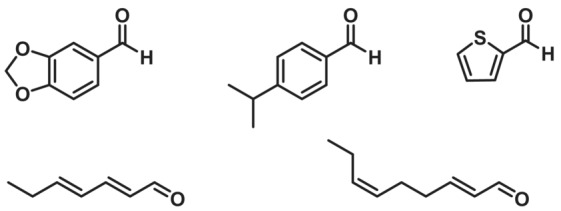
Aldehydes produced by the oxidation of the corresponding primary alcohols by the aryl alcohol oxidase from *Pleurotus eryngii* (*Pe*AAOx2).

Another aryl alcohol oxidase was found in the same fungus (*Pe*AAOx2) and shown to oxidise cumic alcohol and piperonyl alcohol to cuminaldehyde and piperonal with high catalytic efficiencies of 84.1 and 600.2 mM^−1^ s^−1^, respectively.^62^ In a follow‐up study, conditions for a preparative scale reaction were optimized to reach a space–time yield of 9.5 g L^−1^ h^−1^ for piperonal (244.6 mg; 85% yield) and further applied for the oxidation of cumic alcohol, thiophene‐2‐yl‐methanol, (*E*,*E*)‐2,4‐heptadienol and (*E*)‐2‐(*Z*)‐6‐nonadienol (Figure 1). Up to 300 mM of these substrates were converted to the corresponding aldehydes within 20 h.^63^

A new thermotolerant aryl alcohol oxidase from *Moesziomyces antarcticus* (*Ma*AAOx) was recently described as accepting a broad range of primary benzylic alcohols, aliphatic allylic alcohols and furan derivatives like HMF. Among oxidation products were odorous compounds such as benzaldehyde, cuminaldehyde, piperonal and perillaldehyde. Moreover, *Ma*AAOx showed unique activity towards oxidizing 5‐hydroxymethyl‐2‐furancarboxylic acid (HMFCA) to FFCA.^64^

Recently, a high‐throughput screening endeavour identified a new long‐chain alcohol oxidase (LCAOx, EC 1.1.3.20) that is active with a range of fatty alcohols, with 1‐dodecanol being the preferred substrate.^65^

**SCHEME 11:**
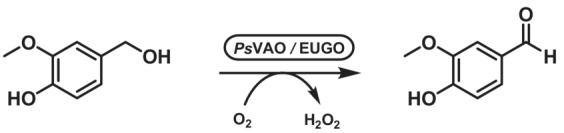
Oxidation of vanillyl alcohol to vanillin catalysed by the vanillyl alcohol oxidase from fungus *Penicillium simplicissimum* (*Ps*VAO) or eugenol oxidase from bacterium *Rhodococcus* sp. strain RHA1 (EUGO).

The VAO family is represented by its name‐giving member: the vanillyl alcohol oxidase, first isolated from the fungus *Penicillium simplicissimum* (*Ps*VAO, EC 1.1.3.38). The fungal flavoenzyme can convert a variety of para‐substituted phenols and produce high‐value aromatic compounds, for example, vanillin and coniferyl alcohol.^66^ Except for alcohol oxidations, the VAO‐type oxidases show a remarkable spectrum of activity: amine oxidations, hydroxylations, ether bond cleavage and even C–C bond formation, which are described elsewhere.^67^ *Ps*VAO is characterized by an autocatalytically formed covalently linked FAD. The prototype reaction is the oxidation of vanillyl alcohol to vanillin (Scheme 11), even though also other substrates can be used as vanillin precursors (e.g. vanillyl amine and creosol). Since fungal VAOs are typically poorly expressed in bacteria, a quest for a bacterial VAO identified a *Ps*VAO homologue in *Rhodococcus* sp. strain RHA1, which could be expressed at high levels in *E. coli*. The bacterial enzyme readily accepted vanillyl alcohol and 5‐indanol as substrates, but the highest activity was observed for the hydroxylation of eugenol to coniferyl alcohol. Therefore, the enzyme was named eugenol oxidase (EUGO).^68^ The optimization of biosynthesis of vanillin using EUGO lead to an organic solvent‐free process with space–time yield of 9.9 g L^−1^ h^−1^, producing 11.2 g of vanillin from a 250 mL scale reaction. Notably, overoxidation to vanillic acid was not detected under the applied conditions.^69^

#### 2.3.2 Alcohol dehydrogenase

**SCHEME 12:**
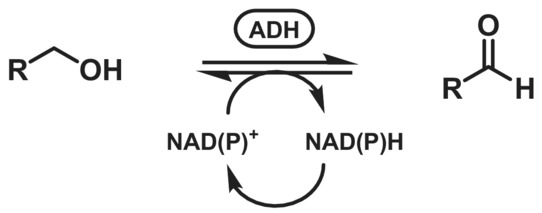
Oxidation of primary alcohols catalysed by alcohol dehydrogenase (ADH).

Alcohol dehydrogenases (ADHs, EC 1.1.1.1) are classical redox catalysts, mediating the reversible transfer of a hydride from an alcohol carbon atom to its associated nicotinamide cofactor (NAD(P)^+^; Scheme 12). Compared to their use in reductive processes (generating chiral centres of secondary alcohols), oxidative applications are far less common.

Alcohol dehydrogenases are the most intensely studied group of oxidoreductases, with numerous known enzymes. The major disadvantage of ADHs is their need for cofactor NAD(P)^+^ which is temporarily loosely bound, relatively unstable and expensive, therefore, a lot of research has been done on efficient cofactor recycling using a molar equivalent of a stoichiometric oxidant (e.g. acetaldehyde or acetone). However, the poor thermodynamic driving force of oxidation reactions often necessitates significant molar surplus of the stoichiometric oxidant in order to drive the reaction equilibrium in the desired direction.^70^ The equilibrium can also be influenced by the pH: usually reduction is preferred at neutral pH, whereas higher pH promotes oxidation.

Despite the large number of known ADHs and studies involving them, the predominant focus is on their use for asymmetric reduction of ketones and enantioselective oxidation of secondary alcohols. Consequently, examples of ADHs applied for aldehyde production are relatively scarce. Overall, many studies reporting expression and characterization of novel ADHs report activity on primary alcohols in the initial substrate screenings, however, often without analysing the oxidation product.^71^

**SCHEME 13:**
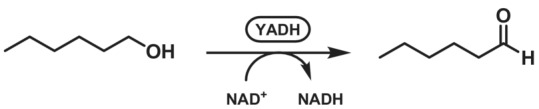
Oxidation of hexan‐1‐ol to hexanal catalysed by alcohol dehydrogenase from *Saccharomyces cerevisiae* (YADH).

From the well‐known ADHs, the horse liver ADH (HLADH), yeast ADH from *Saccharomyces cerevisiae* (YADH) and the ADH from *Geobacillus stearothermophilus* (*Bs*ADH) are known to have broad substrate spectra towards primary alcohols.^31^ HLADH has the best activity with C8 alcohol, with activity detected up to C24,^72^ and was described with, for example, benzyl alcohol^73^ and amino alcohols.^74^ YADH isozymes showed activity with primary unbranched aliphatic C2–C12 alcohols, as well as allyl and cinnamyl alcohol.^75^ Recently, purified YADH was used in a microreactor for production of hexanal reaching volumetric productivity of 4.8 mol L^−1^ day^−1^, without overoxidation that was characteristic for whole cells of *S. cerevisiae* (Scheme 13).^76^ Addition of a second microreactor unit enabled integrated NAD^+^ recycling using the same enzyme with acetaldehyde as the stoichiometric oxidant.^77^

**SCHEME 14:**
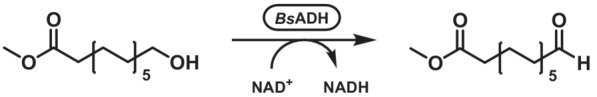
Oxidation of ω‐hydroxy lauric acid methyl ester to ω‐oxo lauric acid methyl ester catalysed by alcohol dehydrogenase from *Geobacillus stearothermophilus* (*Bs*ADH).

The *Bs*ADH is an industrially applicable enzyme that can resist ionic detergents, organic solvents, chaotropic agents, temperatures up to 65°C, but it poorly tolerates oxidative conditions. Recently, it was used for the synthesis of ω‐oxo lauric acid methyl ester (HLAMe) from the corresponding long‐chain alcohol with *K*_M_ = 86 μM and specific activity of 44 U mg^−1^ (Scheme 14). The limited stability of *Bs*ADH under oxidation conditions was improved by a single mutation of an oxidation‐prone residue Cys257 to Leu.^78^

Lyophilized cells of *Janibacter terrae* were used as a catalyst for the chemoselective oxidation of primary alcohols to the corresponding aldehydes with the use of acetaldehyde as the hydrogen acceptor. The substrate spectrum encompassed substituted benzyl alcohols (e.g. producing benzaldehyde and piperonal), *n*‐alkanols and allylic alcohols. A total of 95% conversion of 97 mM benzyl alcohol to benzaldehyde was reported.^73^, ^79^ However, isolation of the responsible ADH was not described to the best of our knowledge.

Propanediol oxidoreductase from *E. coli* (FucO, EC 1.1.1.77) catalyses regioselective oxidation of vicinal diols to produce α‐hydroxy aldehydes. The enzyme was subjected to a directed‐evolution study to access larger aryl‐substituted α‐hydroxy aldehydes.^80^

### 2.4 From amines

The amine functional group is ubiquitous in nature in many forms, such as amino acids and derivatives, amino alcohols, amino sugars and aliphatic mono‐ and diamines. In addition, amine functionalities are crucial in alkaloids, complex nitrogen‐containing structures (often heterocyclic) in plants. Several enzyme classes are very effective in the reversible or irreversible interconversion of primary/secondary amines and the corresponding carbonyls, and such enzymes have been extensively studied and developed. Bioderived primary amines are rare and considered more valuable than aldehydes, so the reverse reaction is much more often applied. Many amine‐converting biocatalysts have become commercially available on large scale, and a few relevant applications will be discussed in this section.

#### 2.4.1 Transaminase/aminotransferase

**SCHEME 15:**
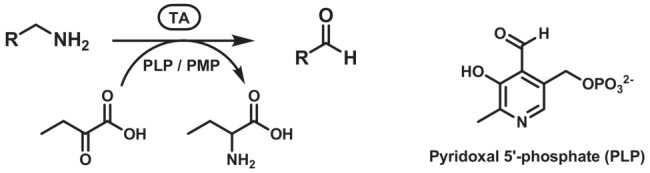
Conversion of primary amines to aldehydes mediated by transaminases (EC 2.6.1.x) and the chemical structure of the cofactor pyridoxal 5′‐phosphate.

Transaminases (TAs, EC 2.6.1.x) also known as aminotransferases (ATs), catalyse the transfer of an amino group from an amino donor to an amino acceptor, which contains a carbonyl group (Scheme 15).^81^ Most commonly, in native enzymes, the transfer occurs between an α‐amino acid and an α‐keto acid, and enzymes with this activity are known as α‐transaminases (α‐TAs). In contrast, there are also enzymes classified as ω‐transaminases (ω‐TAs) which do not require the carboxylic acid functionality to be adjacent to the amine group that undergoes transfer, or which can even accept amines and carbonyl substrates which do not contain a carboxylic acid at all. ω‐TAs are considerably more appealing and useful in synthetic applications because of their broader substrate scope.^82^ TAs have been classified into six subgroups according to the types of substrates accepted and the sequence/structure homologies: classes I–II include most l‐*α*‐TAs (such as l‐alanine TAs and l‐aspartate TAs), class III includes ω‐TAs, class IV includes d‐α‐TAs and branched chain α‐TAs, class V includes l‐serine TAs and class VI sugar TAs.^83^ Transaminases rely on the presence of a pyridoxal 5′‐phosphate (PLP) cofactor (Scheme 15), which is covalently bound to a lysine residue in the active site in the resting state of the enzyme. The typical mechanism is based on a specular sequence of two half‐reactions. Firstly, the amine donor forms an imine with enzyme‐bound PLP, which undergoes de‐protonation and re‐protonation in the active site, forming a different imine, with the C=N bond shifted to the opposite side of the nitrogen atom. Hydrolysis of the latter imine affords the amine version of the cofactor (pyridoxamine 5′‐phosphate, PMP) and the carbonyl compound corresponding to the amine donor, which is released from the enzyme. The second half of the reaction is the mirror image of the first half, where the carbonyl acceptor binds to PMP to form an imine and all steps are reversed to yield the corresponding amine and PLP is regenerated. Kinetic studies demonstrated that PMP is easily lost from the intermediate state, leading often to insoluble and inactive high‐order apoprotein aggregates through intermolecular electrostatic interactions, but the supplementation of extra PLP in solution effectively circumvents the problem.^81^, ^84^, ^85^

The availability of a huge range of well‐characterized and studied wild‐type TAs, as well as broad panels of engineered variants with broadened substrate scope, make those enzymes one of the most efficient and versatile groups of biocatalysts for the synthesis of primary and secondary (often enantiomerically enriched) amines from carbonyls. In spite of the extremely vast literature regarding the use of TAs from laboratory scale to industrial production in this direction, only a small number of examples are reported for the opposite reaction, where the starting material is a terminal primary amine to be converted to an aldehyde.

The formation of aldehydes from a range of hydroxylated arylethylamines has been investigated as a means of supplying such molecules as starting materials or intermediates for multi‐step enzymatic cascades for the synthesis of complex chiral alkaloids, such as benzylisoquinolines and protoberberines.^86^, ^87^ Recently, the applicability of TAs for the production of small aromatic and aryl‐substituted aliphatic aldehydes of interest for the flavour and fragrance industry, starting from the corresponding amines, has been demonstrated in excellent yield (>80%) and purity (>99%). The process was optimized and scaled up using an immobilized TA from the halo‐adapted bacterium *Halomonas elongata*, which was used in a biphasic system under continuous flow with co‐immobilized PLP cofactor.^88^ The same process was also exploited in the production of phenylacetaldehyde and other substituted arylacetaldehydes starting from the corresponding arylethylamines in 90%–95% yield.^89^

Transaminase‐mediated degradation of amino acids is regarded as a key step in the development of flavour in fermented foods, such as cheese and dairy products. Keto acids produced by transamination of amino acids are subsequently degraded to aldehydes or shorter‐chain carboxylic acids and metabolized further.^90^, ^91^ While the addition of isolated TAs is not generally a viable strategy in the food industry, for reasons related to cost and regulatory aspects, the selection of appropriate microorganisms for the process, with their own specific fingerprint of enzymatic activities including TAs, is instrumental to the quality and flavour profile of fermented food.

#### 2.4.2 Monoamine oxidase

**SCHEME 16:**
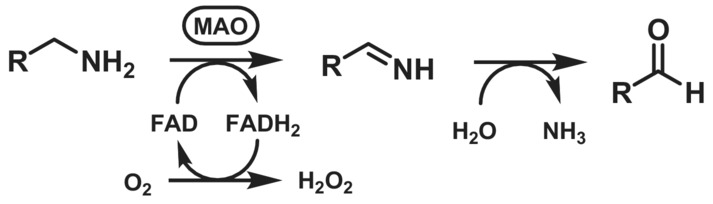
Conversion of primary amines to imines mediated by monoamine oxidase (MAO), followed by spontaneous deamination.

The irreversible oxidation–deamination of primary amines to the corresponding aldehydes, at the expense of the reduction of molecular oxygen to hydrogen peroxide, is catalysed by monoamine oxidases (MAOs, EC 1.4.3.4, Scheme 16).^92^ MAOs are common in mammals, including humans, where they play a key role in the catabolism of food‐derived monoamines (mainly the isoform MAO‐A) and in the metabolism and regulation of monoamine neurotransmitters such as dopamine and norepinephrine, modulating their concentrations in the brain and peripheral tissues (mainly the isoform MAO‐B).^93^ MAO was discovered in 1928 for its ability to oxidise tyramine to *p*‐hydroxyphenylacetaldehyde, and thus originally named tyramine oxidase.^94^

Besides mammalian enzymes, several MAOs have been identified and studied in bacteria and fungi, particularly the homologues present in *Aspergillus niger* (MAO‐N)^95^ and in *Micrococcus luteus*.^96^ MAOs are flavoproteins, with a covalently bound essential FAD prosthetic group.^97^ Due to the richness of the reactivity of the flavin functionality, several mechanisms have been proposed for the oxidative–deamination (single‐electron transfer, step‐wise hydride transfer, two‐step hydride transfer and a polar nucleophilic mechanism).^98^, ^99^ Structurally, MAOs are often characterized by a hydrophobic active site at the bottom of a narrow substrate channel, sometimes with bulky side chains acting as gating residues to control substrate selection and recognition.

The applications of MAO activity to convert primary amines to aldehydes dates back to many decades ago, using partially purified enzyme^100^ or whole cells of *A. niger* overproducing the protein,^101^ for the conversion of dopamine and other biogenic amines. The substrate scope has also been investigated and expanded to the oxidative deamination of a broad range of simple and complex amines.^92^

One of the most powerful drives to the development of engineered MAO variants has been the deracemization of cyclic secondary amines, exploiting an enantioselective MAO for the oxidation and a non‐selective reducing agent.^102^, ^103^ Several of the engineered MAOs thus obtained also showed improved activity against primary and aromatic amines.^104^

MAOs can also be employed for the dehydrogenative synthesis of pyridines and pyrroles.^105^ Lastly, beyond the area of synthetic preparative applications, MAO activity against small benzylamines and arylethylamines is useful for specific analytical assays with aldehydes as intermediates.^106^

#### 2.4.3 Diamine oxidase

**SCHEME 17:**
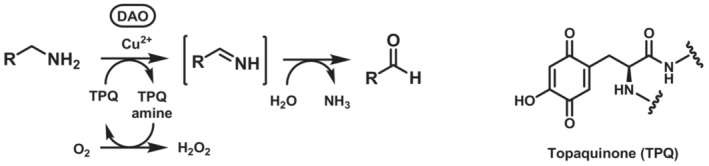
Conversion of primary amines to imines mediated by diamine oxidase (EC 1.4.3.22), followed by spontaneous deamination. The chemical structure of the cofactor topaquinone is also shown.

Diamine oxidases (DAO, EC 1.4.3.22) are isofunctional to MAOs and catalyse oxidative deamination of primary amines at the expense of molecular oxygen, which is reduced to hydrogen peroxide (Scheme 17). DAOs show a very high substrate specificity for linear diamines and polyamines such as putrescine, cadaverine and spermidine, although they were formerly known as histaminases due to their biological role in the degradation of histamine to imidazole acetaldehyde.

Diamine oxidases are homodimeric proteins containing one Cu(II) ion and one trihydroxyphenylalanine quinone (topaquinone, TPQ) molecule in each active site (Scheme 17). The latter is derived from the post‐translational modification of a tyrosine residue in the presence of oxygen, in a spontaneous oxidation reaction mediated by the Cu(II) ion.^107^ The enzymatic reaction involves first the addition of the amine to TPQ followed by dehydration to afford a quinone‐imine intermediate, which undergoes isomerisation and hydrolysis in a similar fashion to the PLP‐dependent transamination mechanism. In the second half‐reaction, the resulting TPQ‐amine undergoes oxidative deamination to regenerate TPQ at the expense of molecular oxygen in the presence of Cu(II).^108^

Human DAO, first produced recombinantly in 2002^109^ has been studied extensively due to its implication in the degradation of histamine linked to food‐related immunological response.^110^ Inhibition studies suggested that an aspartate residue, conserved in all DAOs but not present in other amine oxidase families, is responsible for the specificity towards diamines because it interacts with the second amine group of the most active substrates.

DAOs catalyse the oxidative deamination of many substrates, such as *N*‐alkylputrescines,^111^ triazolyl alkyl amines,^112^ *p*‐chlorophenylethylamine and β‐substituted ethylenediamines.^113^ For instance, a DAO from *Phialemonium* sp. AIU 274 was screened against a panel of amine substrates, showing good conversion of long‐chain alkylamines and aliphatic amino alcohols.^114^ Recently, the DAO isolated from chickpea shoots (*Lathyrus cicera*) gave very high to quantitative conversion with a broad range of amine substrates, and, in order to improve the scalability of the biotransformation, it was purified using a chromatography‐free protocol developed specifically for this enzyme.^115^ The same DAO was also employed in a cascade system to convert the resulting aldehydes into benzylisoquinoline alkaloids.^113^

A relevant area of application of DAO in the food industry is the development of electrochemical biosensors to quantify biogenic amines, linked to food safety issues (spoilage and bacterial contamination) and to potential health problems (allergic reactions or asthma). As representative examples, DAO from *Lathyrus sativus* was applied for electrochemical sensing of biogenic amines in wine and beer samples,^116^ and a DAO from *Arthrobacter crystallopoietes* has been engineered to improve its already high specificity for histamine to be integrated in a biosensor to quantify histamine in fish samples rapidly and inexpensively.^117^

### 2.5 From alkenes

Alkenes are molecules containing at least one C=C double bond and they can be categorised into terminal and internal double bonds. Alkene reagents are essential bulk chemicals and are among the most important raw materials in a plethora of reactions and synthesis processes. Nowadays, most alkenes are produced from petrol and natural gas by processes such as hydrocarbon cracking^118^, ^119^ or from short olefins via the Ziegler Natta^120^ process or the Shell Higher Olefin Process (SHOP).^121^, ^122^ These alkenes can be used, in turn, to produce aldehydes and ketones via an oxidative cleavage. The most relevant alkene‐cleaving enzymes are described in the following sections. Further notable enzymes that are able to catalyse C=C double bond cleavage are: chloroperoxidases^123^ (CPO), horseradish peroxidases (HRP), myeloperoxidase (MPO)^124^ and lignin peroxidases (LiP). It should be noted that alkene cleavage is a side activity of those enzymes.

#### 2.5.1 Non‐heme iron or Mn‐dependent alkene‐cleaving enzymes

Alkene‐cleaving enzymes can be further divided into enzymes using iron as cofactor and non‐iron metal‐dependent alkene cleaving enzymes with, for example, manganese in their active centre as cofactor. The metal ion is chelated in the catalytic centre by amino acid side chains like, for example, four histidines in the case of most carotenoid‐cleaving oxygenases.^125^ Distinct alkene‐cleaving enzymes are described in the following.

##### Carotenoid cleavage oxygenase

**SCHEME 18:**
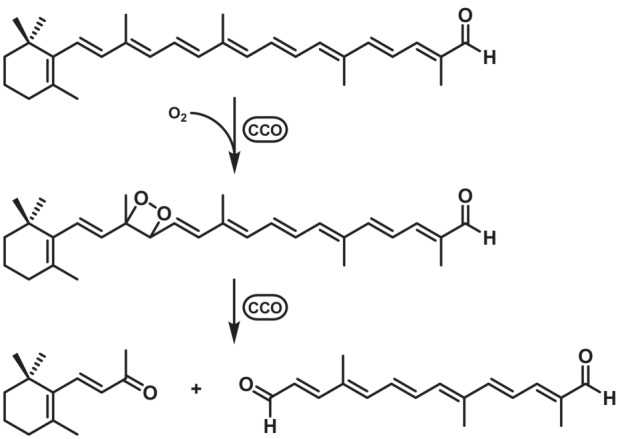
Alkene cleavage of β‐apo‐8′‐carotenal to β‐ionone with a carotenoid cleavage oxygenase/carotenoid cleavage dioxygenase.

Carotenoid cleavage oxygenases (CCOs) or carotenoid cleavage dioxygenase (CCD, both EC 1.13.11.51/67/70/71/82) have their iron cofactor chelated by four histidines in the active site.^125^ A wide substrate scope is reported for this family, spanning from relatively small molecules such as, for example, isoeugenol to large and bulky substrates like their name‐giving natural substrates (carotenoids). As shown in Scheme 18, these enzymes generate dioxetanes as intermediates that decay and give rise to the corresponding aldehydes. Enzymes using this mechanism are considered dioxygenases, since two atoms of oxygen are integrated into the product.^126^ On the other hand, there are CCOs forming an epoxide as an intermediate that can be classified as monooxygenases.^127^

One of the best‐known and most important reactions catalysed by CCOs is the synthesis of vanillin as an essential fragrance and flavour molecule.^128^, ^129^, ^130^ This is usually achieved with a two‐step reaction cascade combined with another enzyme to provide a suitable substrate for the alkene cleavage, such as isoeugenol or 4‐vinylguaiacol, starting from lignin‐derived hydrolytic aromatics.^128^, ^131^, ^132^, ^133^, ^134^ Other interesting products that can be obtained by the direct oxidative cleavage of carotenoids are β‐ionone and dihydro‐β‐ionone, both molecules with a flowery odour. β‐Ionone can be synthesised directly in a one‐step reaction. Dihydro‐β‐ionone is accessible through a one‐pot reaction with a CCO and an additional reductase to reduce the double bond of β‐ionone.^135^ Here, approximately 13.3 mg L^−1^ product was obtained in a one‐pot biosystem, corresponding to over 85% conversion.^135^

Ionones are considered essential fragrance components of scent of tea, grapes, roses, tobacco and wine.^136^, ^137^ CCOs like *At*CCD1 from *A. thaliana* have already attracted interest in biocatalysis for the production of those and other aroma molecules.^123^ Reaction velocity of *At*CCD1 can be increased up to 3.8‐fold using Triton X‐100 as a surfactant to increase substrate solubility^138^ and formation of over 90% yield ionone and perfect regioselectivity was reported in reactions using crude cell lysate.^139^ Furthermore, using lysates with the dioxygenases NOV1 and NOV2, complete conversion of 1 mM of resveratrol and piceatannol were achieved after 20 and 60 min, respectively.^127^

##### Isoeugenol monooxygenase

Isoeugenol monooxygenases (IEMs, EC 1.13.11.88) are often compared to CCOs^133^ since they also chelate an Fe^2+^ between four histidine residues and have generally a similar structure to carotenoid oxygenase. IEMs even possess the same monooxygenase mechanism, as some CCOs first form an epoxide that hydrolyses to the diol before releasing the aldehyde.^128^ It perhaps could be considered to group IEMs to CCOs.

Studies using IEM in combination with a sol–gel chitosan membrane report production of up to 4.5 g L^−1^ (75% conversion) vanillin from 6 g L^−1^ isoeugenol.^140^

##### Lutein cleavage dioxygenase

Lutein cleavage dioxygenase (LCD) is also similar to CCOs where the catalytic site is comprised of an iron ion, coordinated by four histidines. Like IEM, LCDs could also be considered as a subgroup of CCOs or more precisely CCDs.

**SCHEME 19:**
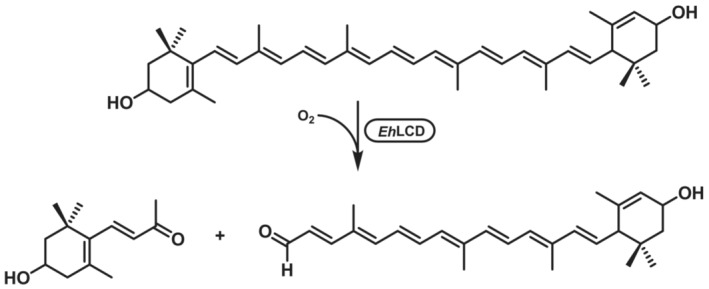
Alkene cleavage of lutein to give 3‐hydroxy‐β‐ionone.

The substrate scope is related to carotenoids, as mainly the name‐giving lutein is converted to 3‐hydroxy‐β‐ionone, a fruity, violet‐like compound.^141^ This is achieved through the cleavage of the double bond between C‐9 and C‐10.^142^ For example with *Eh*LCD from *Enterobacter hormaechei* YT‐3637, 2 mg L^−1^ (87% conversion) product was obtained within 60 min with 1.5 U mL^−1^ of purified enzyme (Scheme 19).^141^ 8‐Methyl‐β‐ionone is another reported product of the degradation of lutein by *E. hormaechei* A20.^143^

##### Cupins

Cupins are a vast family of enzymes containing various different metals as cofactor such as iron, copper, zinc, cobalt, nickel or manganese and are able to catalyse 50–100 different biochemical reactions, for example, isomerisation reactions, hydrolysis or oxygenations. Further exploration and explanation of the diversity of cupins can be found in previous works.^144^, ^145^ Cupins are short enzymes, consisting of 100–150 amino acids on average and four histidines to chelate the metal cofactor. The manganese‐dependent cupin TM1459 from *Thermotoga maritima* is capable to cleave isosafrole and piperine to yield piperonal.^146^ TM1459 is reported to achieve 76% conversion of 4‐chloro‐α‐methylstyrene in biphasic systems using organic hydroperoxide as oxidant. Various other similar styrene derivatives are also reported as accepted substrates.^147^

##### Protease A homologues

The Protease A homologues are a scarcely investigated group of enzymes for C=C cleavage, though members such as AlkCE show promising properties in this field. AlkCE was originally found in the white‐rot basidiomycete *Trametes hirsuta* in 2006. It has a Mn^3+^ centre ion chelated between two aspartic acid residues and one threonine. Unlike most other enzymes, the metal is bound exclusively to oxygen atoms, not to a nitrogen ligand as found in the side chains of histidine.^48^ Alkene‐cleaving reactions can be performed with crude extract and molecular oxygen serves as the sole oxidant. Even freeze‐dried cells exhibit activity on substrates such as *t*‐anethole. Substrates suitable for transformation are 1,2‐dihydronaphthalene, indene, isosafrole as well as several styrene derivatives, and lead to several interesting for flavour and fragrance products such as piperonal or anisaldehyde.^148^

##### Catechol oxygenase

**SCHEME 20:**
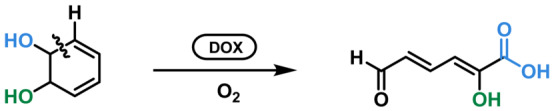
Oxidative ring‐cleavage of catechol to 2‐hydroxymuconaldehyde.

A group of enzymes capable of ring‐opening reactions are catechol oxygenases and they serve as key catalysts in the oxidative degradation of aromatic compounds. These non‐heme iron enzymes use substituted aromatic rings such as catechol as substrate and can be divided into two different classes: intradiol dioxygenases, like catechol 1,2‐dioxygenase (pyrocatechase) and extradiol dioxygenases, such as catechol 2,3‐dioxygenase (also metapyrocatechase) (EC 1.13.11.2). The latter group cleaves the neighbouring double bond of the hydroxy bonds which forms 2‐hydroxymuconaldehyde^123^, ^139^ (Scheme 20) or other derivatives depending on substitutions of the ring. Catechol 2,3‐dioxygenases are Fe(II)‐dependent enzymes with 285 amino acids^149^ that use two histidines and one glutamic acid for coordination of the Fe ion.^139^ Mechanistically, the two oxygens of the substrate are coordinated to the Fe(II), before the oxygen is most likely added by an electrophilic attack, forming a Fe(II)‐semiquinone complex. Then, this leads to a Fe(II) proximal hydroperoxide over a diradical reaction producing an epoxide as an intermediate that undergoes a Criegee rearrangement followed by subsequent hydrolysis to yield the product.^150^, ^151^, ^152^ Using a combination of catechol 2,3‐dioxygenase (cell‐free extract or crude extract) and a bisulfite nucleophilic addition for stabilization of the 2‐hydroxymuconic semialdehyde product, around 75% conversion is achieved starting from 6 g L^−1^ catechol (Scheme 20).^153^

##### Glyoxal‐forming diketone‐cleaving enzyme

**SCHEME 21:**
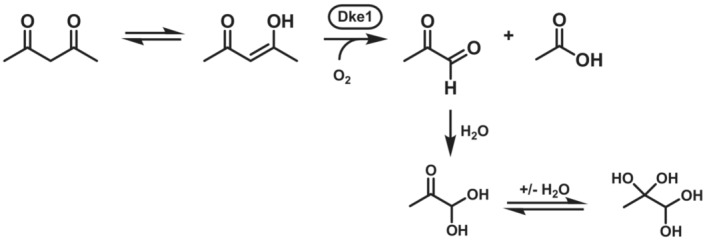
Cleaving reaction of diketone‐cleaving enzyme.

Diketone‐cleaving enzyme (Dke1; EC 1.13.11.50) is a 153 amino acid long enzyme that is capable to cleave 2,4‐pentanedione (acetylacetone) to acetate and methylglyoxal with the consumption of one equivalent of molecular oxygen.^154^, ^155^ The enzyme is very specific, since the β‐diketone has to be present in its enol for the addition of oxygen as depicted in Scheme 21. Polar aldehydes such as methylglyoxal tend to be hydrolysed in aqueous solution. Examples of substrates that can be consumed are 2,4‐octanedione, 2‐acetylcyclohexanone and 3,5‐heptanedione. The presence of two ketones in beta position are essential, since their exchange to other moieties such as alcohols or carboxylic acids results in no conversion.^154^ The Fe^2+^ cation in Dke1 serving as the catalytic centre, is bound by a triad consisting of histidines^156^, ^157^, ^158^ instead of the 2‐His‐1‐carboxylate facial triad commonly found in other mononuclear non‐heme iron enzymes.

#### 2.5.2 Heme‐dependent alkene‐cleaving enzymes

Heme‐dependent alkene cleaving enzymes possess ferric protoporphyrin IX or protoheme and are capable of using molecular oxygen or hydrogen peroxide as the oxidant. Additionally to the heme complex, the iron can be coordinated by a histidine or serine from below the plane of the porphyrin. Two examples for oxygen‐requiring enzymes that are able to catalyse oxidative ring cleavage are tryptophan 2,3‐dioxygenase (TDO) and indoleamine 2,3‐dioxygenase (IDO). Their substrates scope is wide and varies from cyclic systems to polymers such as rubber.

##### Tryptophan 2,3‐dioxygenase and Indoleamine 2,3‐dioxygenase

**SCHEME 22:**
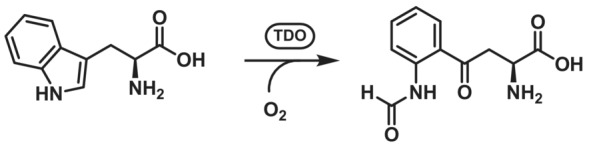
Oxidative ring‐cleaving reaction catalysed by tryptophan 2,3‐dioxygenase.

Tryptophan 2,3‐dioxygenases^159^ (TDO, EC 1.13.11.11) and indoleamine 2,3‐dioxygenases^160^, ^161^ (IDO, EC 1.13.11.17) are classified as heme‐dependent alkene‐cleaving enzymes and use molecular oxygen for a ring‐opening reaction via the cleavage of a C=C double bond. Both serve in tryptophan catabolism through the kynurenine pathway and are also produced in cells in response to inflammation. They are especially highly expressed in tumour cells to reduce detection by the immune system.^162^, ^163^ TDOs are 35–45 kDa proteins and are tetramers, while IDOs are monomeric enzymes with an approximate mass of 45 kDa. Though isofunctional, they possess a low sequence identity with 16% between human TDO and human IDO1.^161^ An important difference in the sequence is the presence of a histidine that interacts with the NH group of the indole ring of l‐tryptophan, aiding in substrate binding in TDOs, while IDOs lack this residue and have a serine in this place.^164^ Heme iron serves to activate the oxygen to allow the rection to occur. Nowadays, a radical addition mechanism is postulated as the first step,^161^ but alternative mechanisms for the initial oxygen addition have also been suggested.^165^ Over a peroxo transition state, an epoxide intermediate and a ferryl heme [Fe(IV)] are formed. The presence of a ferryl heme was confirmed in IDOs.^166^, ^167^, ^168^ Protonation opens the epoxide ring and triggers the addition of oxygen chelating the iron to C2, leading to the formation of *N*‐formyl‐l‐kynurenine (Scheme 22).^161^ Both TDO and IDO have already been shown to be able to completely convert several substrates such as tryptophan at low concentrations.^123^

##### Rubber oxygenases RoxA and RoxB

Rubber oxygenases (Rox, EC 1.13.11.x) are extracellular c‐type diheme dioxygenases^169^ found in rubber‐degrading bacteria that can cleave the C=C double bond of poly(*cis*‐1,4‐isoprene). While the cleavage product of RoxA is mainly 2‐oxo‐4,8‐dimethyltrideca‐4,8‐diene‐1‐al (ODTD), a C_15_ oligoisoprenoid,^170^ RoxB possesses a distinctive product spectrum of C_20_, C_25_, C_30_ and higher oligo‐isoprenoids.^171^ RoxA consists of 678 amino acids and features two binding sites for covalent attachment of heme.^137^, ^172^ The reaction is performed without additional cofactors and uses O_2_ as oxidant. First, the double bond attacks the oxygen that deprotonates the substrate, forming temporarily a hydroperoxide and shifting temporarily the double bond between C1 and C2. Then, spontaneously, a 1,2‐dioxetane is formed as an intermediate that releases the corresponding aldehydes upon rearrangement.^169^

### 2.6 From cyanohydrins

α‐Hydroxynitriles (alternative name: cyanohydrins) occur in plants in the form of cyanogenic glycosides for the purpose of defence against herbivores. Approximately 25 cyanogenic glycosides are known in plants, whereby the cyanohydrin moiety is attached to a mono‐ or disaccharide.^173^ Also, certain insects harbour such glycosides.^174^

#### 2.6.1 Hydroxynitrile lyase

Hydroxynitrile lyases (HNL; EC 4.2.1.X) catalyse the cleavage of cyanohydrins, which are released from cyanogenic glycosides by glycosidases in the first step.^175^ In this manner, simple aldehydes like benzaldehyde and isobutyraldehyde are accessible from renewable sources. For synthetic purposes, the reverse reaction is evidently much more relevant, as it affords a chiral, bi‐functional molecule, which can undergo a great variety of follow‐up reactions.^176^, ^177^ In this sense, HNLs have considerably more impact in aldehyde transformation than they do in aldehyde synthesis.

### 2.7 From hydroperoxides and epoxides

Hydroperoxides and epoxides are formed in nature by reaction of unsaturated compounds such as fatty acids with reactive oxygen species in an untargeted manner or with metal‐dependent redox enzymes in a targeted fashion, and they play a major role in redox‐dependent signalling. Reactive epoxides emerge in the degradation of aromatic compounds.^178^ Plants, for example, utilize polyunsaturated fatty acids as substrates for lipoxygenases (LOX)^179^, ^180^ or α‐dioxygenases (αDOX), which produce fatty acid hydroperoxides as precursor molecules for aldehydes that are formed in a second step and often give rise to the typical odour of the respective plant.

#### 2.7.1 *α*‐Dioxygenase

**SCHEME 23:**
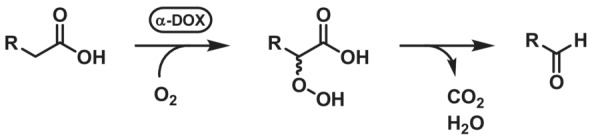
α‐Dioxygenase‐mediated fatty acid hydroperoxidation followed by spontaneous decarboxylation to yield carbon chain shortened fatty acid.

α‐Dioxygenases (α‐DOXs, EC 1.13.11.92) oxidise medium‐chain fatty acids to 2‐hydroperoxy fatty acids (Scheme 23). Spontaneous decarboxylation and dehydration give rise to carbon chain‐shortened fatty aldehydes and one equivalent each of H_2_O and CO_2_.^181^ The substrate scope of α‐DOXs differs depending on their origin. Plant enzymes accept longer chain fatty acids (>C_14_), whereas cyanobacterial α‐DOXs studied up to date show preference for shorter chain fatty acids (C_10_–C_16_).^182^, ^183^, ^184^ From the mechanistic viewpoint, α‐DOXs are heme‐dependent and carry an essential tyrosine residue close to the heme. Similar as in CRO‐AOx, a tyrosyl radical is formed. The α‐hydrogen of the fatty acid substrate is transferred to the tyrosyl radical, followed by O_2_ radical trapping and non‐enzymatic decarboxylation of the formed α‐hydroperoxy fatty acid.^185^ For more comprehensive information about α‐DOXs and its applications for fatty aldehyde synthesis, the reader is referred to a very recent review.^185^ Notably, some applications target the formation of a mixture of aldehydes. In a recent example, an α‐DOX was coupled to a fatty aldehyde dehydrogenase, which lead to the iterative decarboxylation of long‐chain fatty acids to mixtures of long‐ and medium‐chain aldehydes.^186^

#### 2.7.2 Hydroperoxide lyase

**SCHEME 24:**
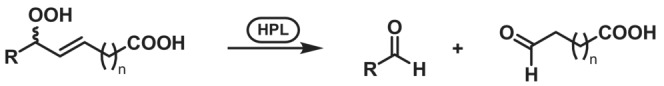
Hydroperoxide lyase mediated isomerisation of fatty acid hydroperoxides to short‐lived hemi‐acetals, followed by spontaneous hydrolysis into two aldehyde species.

Hydroperoxide lyases (HPLs, EC 4.1.2.x) are known to catalyse the cleavage of hydroperoxides of polyunsaturated fatty acids to a short‐chain aldehyde and an ω‐oxo acid respectively. HPLs are a particular subclass of heme proteins of the cytochrome P450 family (CYP74). They do not function as monooxygenases, but isomerize the hydroperoxide into a short‐lived hemi‐acetal,^187^ which finally decomposes to two aldehyde species. In plants, HPL is acting in concert with lipoxygenases of matching product selectivity, depending on the (poly)unsaturated fatty acid that is available as a substrate.^180^ In essence, the overall reaction sequence resembles that of alkene‐cleaving enzymes (Section 2.5). Typical substrates of LOX/HPL cascades are linoleic acid, α‐linolenic acid, oleic acid and arachidonic acid. In context of arachidonic acid metabolism, cyclooxygenase (COX) is worth to be mentioned as a producer of hydroperoxyl arachidonic acid.^188^ Depending on the regio‐ and stereoselectivities of the enzymes, green leaf volatiles (e.g. C_6_, C_9_) are formed (Scheme 24). On analytical level, aldehyde production with recombinant HPL in *E. coli* appears to be promising.^189^ When utilizing the LOX/HPL system recombinantly in microbial hosts, the aldehydes are often (intentionally) metabolized further, hence, mostly alcohol formation has been published in synthetic applications. In a recent example, tomato HPL was expressed in *E. coli* and used for (*Z*)‐3‐hexenol synthesis.^190^ Impressive space–time yields of >8 g L^−1^ h^−1^ were published for the same product in non‐optimized batch reactions using bacterial cell suspensions with engineered guava HPL in combination with a commercial ADH for aldehyde reduction.^191^

#### 2.7.3 Styrene oxide isomerase

**SCHEME 25:**
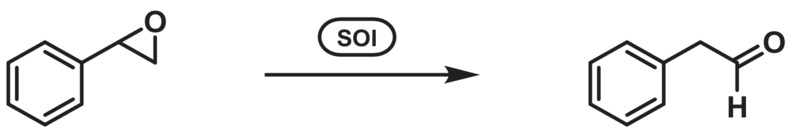
Name‐giving reaction of styrene oxide isomerases. The reaction resembles the Meinwald rearrangement.

Styrene oxide isomerase (SOI, EC 5.3.99.7) catalyses the isomerisation of styrene oxide to phenylacetaldehyde (Scheme 25).^192^ In case of *trans*‐1‐phenylpropylene oxide, the terminal methyl group undergoes a 1,2 shift and enantiomerically pure 2‐phenylpropanal is formed. The stereochemistry of the product depends on the absolute configuration of the epoxide.^193^ The substrate scope of SOIs appears to be quite restricted to epoxides adjacent to a phenyl (styrene and a handful of substituted styrene derivatives).^194^ SOIs are small (20 kDa) membrane‐bound proteins, which might be the reason that a protein structure is not available yet, and also the reaction mechanism remains largely elusive.^194^ Nevertheless, the enzyme finds application in various cascade reactions,^194^ the production of phenylacetaldehyde (vide infra)^195^ and 2‐phenylethanol production.^196^

#### 2.7.4 Benzoyl‐CoA dihydrodiol lyase (BDL)

**SCHEME 26:**
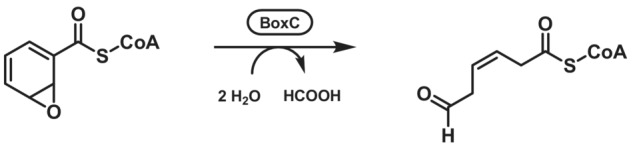
Aldehyde‐forming step in anaerobic benzoate degradation.

Benzoyl‐CoA dihydrodiol lyase (BoxC; EC 4.1.2.44) catalyses the hydration of the non‐aromatic 2,3‐epoxide of benzoyl‐CoA that is a metabolite in the anaerobic degradation of benzoate. Two molecules of H_2_O are required to generate 3,4‐dehydroadipyl‐CoA semialdehyde and one equivalent of formate (Scheme 26).^197^ Relevance of this reaction in a synthetic context has not been demonstrated yet.

### 2.8 From keto acids

Keto acids are essential organic compounds that contain a keto group and a carboxyl group.^198^ They have different natural functions, and their stability depends on the position of the carbonyl group. Currently, the majority of α‐keto acids are produced by chemical synthesis, but their biotechnological production, mainly biotransformation, and de novo synthesis from inexpensive renewable carbohydrates are promising alternatives.^199^, ^200^, ^201^ Therefore, α‐keto acids are used as a source of aldehyde precursors in non‐oxidative decarboxylation in which a corresponding aldehyde and carbon dioxide are formed.^202^

#### 2.8.1 Pyruvate decarboxylase

**SCHEME 27:**
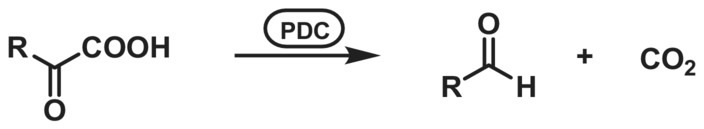
Decarboxylase‐mediated disintegration of α‐oxoacids to aldehyde and carbon dioxide.

Pyruvate decarboxylase (PDC; EC 4.1.1.1) is an extensively examined class of decarboxylases, involved in the ethanol production pathway. PDCs are prevalent in yeast and plants and are seldom present in prokaryotes. Their primary function is to decarboxylate pyruvate to acetaldehyde and carbon dioxide (Scheme 27).^203^ Moreover, PDCs are able to utilize other aliphatic as well as aromatic 2‐oxoacids. The catalytic activity of PDC depends on the presence of the cofactor thiamine diphosphate (ThDP), which is bound mainly via a Mg^2+^ ion to the protein moiety at the interface of two subunits.^204^

Since acetaldehyde is an inhibitory and toxic by‐product for microorganisms, intracellular concentrations are typically low. However, *Lactobacillus lactis* was engineered to accumulate high yields of acetaldehyde (0.418 g L^−1^) produced by decarboxylation of pyruvate by PDC originating from *Z. mobilis*.^205^ Not only *L. lactis* but also *E. coli* was engineered to overproduce acetaldehyde starting from glucose. Acetaldehyde concentration was doubled to 0.725 g L^−1^ by knocking out the competing metabolic pathways.^206^ Acetaldehyde is not only a valuable compound used as a substrate for further reactions, but also an important aroma in yoghurts and other dairy products.^205^

#### 2.8.2 Other α‐keto acid decarboxylases

Benzoylformate decarboxylase (BFD, EC 4.1.1.7) was originally isolated from *Pseudomonas putida* but was also purified from *Acinetobacter* species. These enzymes are also ThDP‐Mg^2+^‐dependent with certain similarities to *Zm*PDC. BFD plays an inevitable role in the mandelate pathway, where it catalyses the removal of CO_2_ from benzoyl formate to generate benzaldehyde.^207^ Benzaldehyde is one of the most frequently applied aromas in the food industry. It is interesting due to its bitter almond odour with cherries, malt or roasted pepper tones.^208^ However, BFD is also active towards certain aliphatic α‐keto acids, such as pyruvic acid, 2‐oxobutyric acid, 4‐methyl‐2‐oxopentanoic acid that are converted at lower rates. The products of these substrates are acetaldehyde, propanal and 3‐methylbutanal, respectively.^209^

Branched‐chain keto acid decarboxylases (KdcAs, EC 4.1.1.72) have been found in different *L. lactis* strains. The substrate scope of KdcAs is wider in comparison to PDCs, but both enzymes are ThDP‐dependent.^210^ KdcA is involved in the Ehrlich pathway, which eventually leads to the formation of alcohols from branched‐chain amino acids.^211^ In addition to the main pathway, amino acid catabolism may result in hydroxy acids, esters and likewise in high‐impact aldehyde flavours formed from valine, isoleucine, phenylalanine and from other amino acids.^212^, ^213^ Other aldehydes accessible through KdcA‐mediated decarboxylation worth mentioning are methional (potato flavour present in crisps) or phenylacetaldehyde with a sweet, honey‐like odour resembling hyacinths and frequently added to perfumes.^1^

Ketoisovalerate decarboxylase (KID) is the second decarboxylase isolated from *L. lactis* with almost 90% amino acid sequence identity with KdcA.^212^ Therefore, KID also participates in the metabolism of amino acids, where it is involved in the production of 3‐methylbutanal starting from leucine.^214^ 3‐Methylbutanal is the aroma with caramel cocoa flavour, which contributes to the nutty flavour of Cheddar cheese.^214^, ^215^ Various strains of *L. lactis* were isolated from fermented products and tested for the activity of KID. The highest yields showed strain 408 that produced 12.1 ± 0.6 mg L^−1^ of 3‐methylbutanal.^216^, ^217^ Alternatively, recombinant KID was produced together with leucine dehydrogenase for in vitro cascade reactions from valine to 3‐methylbutanal. In continuous mode, the best catalyst formulation gave 340 mg L^−1^ product concentration.^218^

### 2.9 From other precursors

#### 2.9.1 Chloroacrylic acid dehalogenase

**SCHEME 28:**
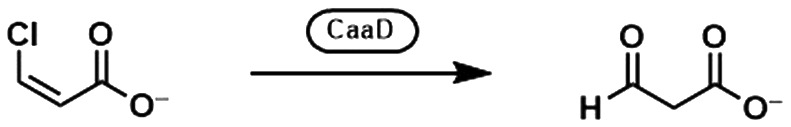
Tautomerase catalysed dehalogenation.

Chloroacrylic acid dehalogenases (CaaDs) are tautomerase enzymes that catalyse the hydrolytic dehalogenation of *cis*‐3‐chloroacrylic acid to afford malonate semialdehyde (Scheme 28).^219^

## 3 FLAVOUR AND FRAGRANCE ALDEHYDES TARGETED BY ENZYMATIC REACTIONS

### 3.1 Aromatic aldehydes

Vanillin is the spotlight aldehyde and various routes to this compound have been explored in academia and industry, up to production level.^220^ Biocatalytic vanillin production from ferulic acid was, for example, commercialized more than 20 years ago using wild‐type organisms.^5^ The most recent report shows vanillin formation from isoeugenol with a recombinant isoeugenol monooxygenase (Jin1 from *Pseudomonas nitroreducens*) at 0.3 M substrate load in a space–time yield of 4.8 g L^−1^ h^−1^.^221^

Piperonal (heliotropin) is an aromatic aldehyde that is structurally related to vanillin. It is a floral‐type odour with creamy cherry and vanilla smell.^136^ This aldehyde was enzymatically prepared from piperonylic acid with CAR as the key enzyme in an *E. coli*‐based living cell catalyst with as space–time yield of 1.5 g L^−1^ h^−1^.^222^ Starting from the respective alcohol, space–time yield of 9.5 g L^−1^ h^−1^ was achieved using AAOx.^63^ In both reports, piperonal was isolated in high yield and >99% purity via simple crystallization from *n*‐hexane extracts.

Further benzaldehyde derivatives are accessible through the action of monooxygenases via oxidation of their primary products, the respective alcohols. The non‐heme iron monooxygenase XylM, for example, was used for the production of 3,4‐dimethylbenzaldehyde from pseudocumene in a space–time yield of 1.6 g L^−1^ h^−1^ after systematic optimization.^223^ One of the key tasks here was the suppression of further oxidation to the respective acid and this was—amongst others—achieved by in situ removal of the aldehyde, the most frequently used strategy in bioaldehyde production.

### 3.2 Aliphatic aldehydes

Acetaldehyde is a short‐chain aldehyde with a pungent, ethereal, fresh and fruity odour. This aldehyde inhibits microbial growth at millimolar concentrations and therefore is a rather challenging fermentation product. *Zymomonas mobilis* shake flask cultivation gave up to 1.6 g L^−1^ acetaldehyde with its native PDC.^224^ The AOx of *Pichia pastoris* was used in biotransformation mode with resting cells and delivered up to 70 g L^−1^ of acetaldehyde when ISPR with TRIS was used.^225^

The odour perception of isobutyraldehyde is described as fresh, aldehydic, floral, pungent and green. Rodriguez and Atsumi engineered *E. coli* towards isobutyraldehyde production using a *L. lactis* keto acid decarboxylase (KID) for the aldehyde‐forming step from 2‐ketoisovalerate. To suppress further reduction to isobutanol, altogether 15 genes were knocked out. The final strain reached a product titre of 35 g L^−1^ after 5 days (space–time yield of 0.29 g L^−1^ h^−1^), using gas stripping as ISPR strategy.^226^

Hexenal and hexanal are referred to as typical ‘green leaf volatiles’ (GLVs) because their smell is associated to freshly cut grass, green fruits and vegetables. Traditionally, plant‐derived enzyme preparations were used for GLV production from polyunsaturated fatty acids: Akacha and Gargouri, for example, used olive leaf‐derived HPL to produce hexenal in a liquid/gas reactor and achieved 50% hexenals with respect to the added substrate 13‐(*S*)‐hydroperoxylinolenic acid, which corresponded 0.36 g kg^−1^ of reaction medium.^227^ Recombinant, engineered guava HPL was published by Firmenich SA for green leaf alcohol synthesis (8 g L^−1^),^191^ and with further optimization is likely an applicable biocatalyst for production scale of both, GLV aldehydes and alcohols.

The odour of octanal is described as waxy, fatty, citrus, orange, peely, green, herbal, aldehydic and fresh. High aldehyde yield was achieved for the conversion of octanoic acid to octanal using a recombinant CAR from *Mycobacterium marinum* in engineered *E. coli* cells in the presence of *n*‐heptane as ISPR solvent.^228^ When opting for this strategy, ISPR has the double function of protecting the aldehyde product from cell‐mediated follow‐up reactions and protecting the cells from the cytotoxic product compound. Biocompatible solvents like hexadecane hold even more promise, as they are able to sustain cell viability and therefore ATP supply even better.

### 3.3 Aryl‐aliphatic aldehydes

Phenylacetaldehyde is a green‐type odour with a sweet and floral smell, also reminding of honey and cocoa/chocolate. This aldehyde can be produced from styrene with SOI. High productivity of this isomerase enzyme was showcased by attaching a small protein fusion tag to enhance its expression, resulting in the highest reported phenylacetaldehyde production of (405 g L^−1^) from styrene oxide so far.^195^

The spicy odour type cinnamaldehyde is perceived as cinnamon, sweet, aldehydic, aromatic, balsamic, resinous and powdery, sometimes also honey. Cinnamaldehyde was in the spotlight of a recent study which utilised an engineered cinnamic acid producing *Corynebacterium glutamicum* strain. The same *C. glutamicum* strain was equipped with *Mycobacterium phlei* CAR (*Mp*CAR). Undesired aldehyde metabolism was suppressed by deletion of four genes. Cinnamaldehyde (2.2 g L^−1^ h^−1^) was obtained after full conversion of crude cinnamic acid (1.2 g L^−1^) from a *C. glutamicum* culture supernatant.^229^ Higher product titres were obtained in the laboratory by a heavily engineered *E. coli* strain in combination with *Mm*CAR, however, space–time yields were considerably lower (0.049 g L^−1^ h^−1^).^230^

### 3.4 Heteroaromatic aldehydes

Even though furfural and 5‐hydroxymethylfurfural (HMF) belong to the most abundant biomass‐derived aldehydes, there is also research on their synthesis using chemoenzymatic pathways including glucose isomerase^231^ and microbial biosynthesis by introducing heterologous pathways into microorganisms.^232^ Furthermore, oxidation of HMF by AOx was discussed in Section 2.3.1.

**SCHEME 29:**
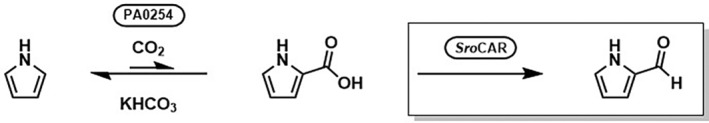
One‐pot production of pyrrole‐2‐carbaldehyde from pyrrole, with a carboxylation performed with a UbiD‐type decarboxylase from *Pseudomonas aeruginosa* (*PA*0254) and CAR from *Segniliparus rotundus* (*Sro*CAR) as a whole‐cell biocatalyst.

Pyrrole‐2‐carbaldehyde is a musty‐type odour found in the highest concentration not only in beer but also in teas and coffees. Synthesis from pyrrole and CO_2_ was reported using a one‐pot system with a UbiD‐type decarboxylase from *Pseudomonas aeruginosa* HudA/PA0254 in combination with CAR from *Segniliparus rotundus* (*Sro*CAR) as a whole‐cell biocatalyst (Scheme 29). Low maximum product yield of 2.14 ± 0.16 mM was assigned to the instability of the target product. Except pyrrole‐2‐carboxylic acid, the *Sro*CAR was capable of reducing furan‐ and thiophene‐2‐carboxylic acids to their corresponding aldehydes.^233^ A similar two‐step approach (using a decarboxylase from *Arthrobacter nicotianae* and CAR from *S. rugosus*) was used to produce indole‐3‐carbaldehyde from indole.^234^

## 4 CONCLUSIONS

Aldehydes are challenging chemicals to make, due to their high reactivity. In many cases, there is a kinetic preference for aldehyde reactions in comparison to the reactivity of their precursor molecules. This is a fact in chemical as well as in enzymatic or biocatalytic production of aldehydes. Herein, we described enzymatic routes from 11 classes of precursor molecules that may be treated with enzymes to produce aldehydes as their products. A 12^th^ possibility was omitted deliberately, which is the formation of aldehydes by aldolases, which interconvert one aldehyde into another. While some of the presented reactions may be perceived as laboratory curiosities, others have certainly the potential to be used for synthesis (Table 1). We devoted a chapter to those reactions which delivered the best yields of molecules perhaps most relevant for the flavour and fragrance sector. In this respect, the most often selected reactions are alcohol oxidation by alcohol oxidases, acid reduction by carboxylic acid reductases, keto acid decarboxylation by decarboxylases and the combination of lipoxygenases with hydroperoxide lyases. In terms of catalyst form, the most prevalent formulations are resting cell or living cell biocatalysts. On the one hand, this is a necessity in case metabolic reactions are required, and on the other hand, it is owed to handling simplicity and enzyme stability.

**TABLE 1:** An overview of biocatalytic flavour and fragrance aldehyde‐forming reactions.

Products	Substrates	Enzymes	Catalyst form	Volumetric productivity or STY	Reference
Vanillin	Isoeugenol	IEM	Resting cells	4.8 g L^−1^ h^−1^	221
Piperonal	Piperonylic alcohol	AAOx	Purified enzyme	9.5 g L^−1^ h^−1^	63
Piperonal	Piperonylic acid	CAR	Resting cells	1.5 g L^−1^ h^−1^	222
3,4‐Dimethylbenzaldehyde	Pseudocumene	AOx	Living cells	1.6 g L^−1^ h^−1^	223
Acetaldehyde	Pyruvate	PDC	Living cells	1.6 g L^−1^ (10 h)	224
Acetaldehyde	Ethanol	AOx	Resting cells	44 g L^−1^ (24 h)	225
Isobutyraldehyde	2‐ketoisovalerate	KID	Living cells	35 g L^−1^ (5 days)	226
Hexenal	13‐Fatty Acid Hydroperoxides	HPL	Cell lysate	8 g L^−1^ h^−1^	191
Octanal	Octanoic acid	CAR	Living cells	0.458 g L^−1^ h^−1^	228
Phenylacetaldehyde	Styrene oxide	SOI	Resting cells	405 g L^−1^ (2 h)	195
Cinnamaldehyde	Cinnamic acid	CAR	Living cells	2.2 g L^−1^ h^−1^	229
Pyrrole carbaldehyde	Pyrrole‐2‐carboxylic acid	CAR	Living cells	0.2 g L^−1^ (18 h)	233

## CONFLICT OF INTEREST STATEMENT

The authors declare no financial or commercial conflict of interest.

## Data Availability

Data sharing is not applicable to this article as no new data were created or analyzed in this study.
